# Cardiovascular Risk Assessment in Psychiatric Patients

**DOI:** 10.1192/j.eurpsy.2025.1539

**Published:** 2025-08-26

**Authors:** J. Barreira, S. Macedo

**Affiliations:** 1 Psychiatry and Mental Health, Unidade Local de Saúde do Tâmega e Sousa, Porto, Portugal

## Abstract

**Introduction:**

Psychiatric patients, particularly those with severe mental illness, face an elevated risk of cardiovascular disease. This heightened risk stems from a combination of factors, including the effects of antipsychotic medications, unhealthy lifestyles, and socioeconomic disadvantages. Addressing cardiovascular risk in this population requires tailored approaches that consider both medical and mental health aspects. Traditional cardiovascular risk prediction models, such as SCORE2, often fail to accurately assess cardiovascular risk in psychiatric patients.

**Objectives:**

This non-systematic review aims to evaluate the evidence behind the use of cardiovascular risk prediction models in patients with mental illness, assessing their applicability and limitations.

**Methods:**

Relevant and recent studies or reviews were selected from the PubMed electronic database using search terms related to cardiovascular risk prediction and mental illness. Articles were chosen based on their relevance to psychiatric populations and their focus on prediction models.

**Results:**

Recent cardiovascular risk prediction models, such as PRIMROSE and QRISK3, incorporate factors like the presence of severe mental illness, antipsychotic and antidepressant use, and lifestyle factors (smoking and alcohol consumption). These models are more effective than general population models in predicting cardiovascular events in psychiatric patients. Interventions based on these models, particularly pharmacological strategies such as the use of statins and metformin, have been effective in managing lipid levels and reducing cardiovascular risk in this population.

**Conclusions:**

Psychiatric patients are at a significantly increased risk of cardiovascular disease, warranting early intervention and consistent monitoring. Tailored risk assessment models can significantly improve cardiovascular outcomes by guidind pharmacological intervention. Further studies are needed to improve and validate the available prediction models.

**Disclosure of Interest:**

None Declared

